# Retrospective Study of Energy Requirement Recommendations for Dogs in a Brazilian Veterinary Hospital (2013–2025)

**DOI:** 10.3390/ani15152226

**Published:** 2025-07-29

**Authors:** Pedro Henrique Marchi, Leonardo de Andrade Príncipe, Gabriela Luiza Fagundes Finardi, Natália Manuela Cardoso de Oliveira, Gabriela Pinheiro Tirado Moreno, Maria Carolina Farah Pappalardo, Felipe Sesti Trindade, Júlio Cesar de Carvalho Balieiro, Thiago Henrique Annibale Vendramini

**Affiliations:** 1 Pet Nutrology Research Center (CEPEN Pet), Department of Animal Nutrition and Production, School of Veterinary Medicine and Animal Science, University of Sao Paulo, Pirassununga 13635-900, Brazil; pedro.henrique.marchi@usp.br (P.H.M.); leoprincipe@usp.br (L.d.A.P.); gabriela.finardi@usp.br (G.L.F.F.); felipesestitrindade@usp.br (F.S.T.); balieiro@usp.br (J.C.d.C.B.); 2 Veterinary Nutrology Service, Veterinary Teaching Hospital, School of Veterinary Medicine and Animal Science, University of Sao Paulo, Sao Paulo 05508-270, Brazil; nataliamanuela@usp.br (N.M.C.d.O.); gabrielapinheirotm@gmail.com (G.P.T.M.); carolpappalardo@gastropet.com.br (M.C.F.P.)

**Keywords:** energy intake, metabolism, neutering, nutritional assessment

## Abstract

Accurate estimation of metabolizable energy requirements (MER) is essential for appropriate dietary planning in dogs. However, standardized recommendations may not reflect the variability observed in clinical practice, particularly regarding individual factors such as neutering status, body condition, and age. This study aimed to retrospectively assess the MER factor in dogs treated at a Brazilian veterinary hospital, considering variables including sex, neuter status, body condition score (BCS), muscle mass score (MMS), body size, and life stage, and to compare the findings with those typically reported in the scientific literature. A total of 438 medical records from 2013 to 2025 were reviewed for this study. Data on demographic and clinical variables were collected, and MER factors were calculated based on energy intake and body weight. Statistical analyses were conducted to evaluate the interactions between variables, particularly BCS and neutering status. The study population included 253 males (57.8%) and 185 females (42.2%), with 80.6% of them being neutered. The MER values varied widely across the subgroups. Neutered dogs had consistently lower energy factors than intact dogs (*p* < 0.0001), and energy requirements decreased progressively with increasing BCS, regardless of neutering status (*p* < 0.05). A significant interaction between BCS and neutering status was observed (*p* = 0.0089), although BCS alone was not a significant predictor (*p* = 0.9670). No substantial differences were observed based on sex, body size, or life stage. MER values observed in clinical practice differ from standard recommendations and are influenced by the interaction between neutering status and body condition. These findings reinforce the need for individualized nutritional assessments in companion animal medicine and call for further studies to refine clinical energy estimation models.

## Simple Summary

To feed dogs correctly, it is necessary to know how much energy they require each day. However, the standard energy recommendations found in textbooks and guidelines may not always match those observed in real-life clinical settings. In this study, we analyzed the medical records of 438 dogs treated at a veterinary hospital in Brazil over a 12-year period. We found that energy needs varied depending on factors like whether the dog was neutered and its body condition. Neutered dogs required less energy than intact dogs, and overweight or obese dogs required less energy per kilogram of body weight than dogs in ideal condition. These results suggest that veterinarians should consider each animal’s individual characteristics when calculating daily energy requirements rather than relying only on general recommendations.

## 1. Introduction

Although not a nutrient itself, energy is essential for all biochemical and metabolic functions in dogs. Adult pet dogs require a specific amount of daily energy, which is determined by energy expenditure, which includes the basal metabolic rate, voluntary muscle activity, caloric increment, and thermogenesis [1]. The basal metabolic rate corresponds to the minimum energy required to maintain the body’s vital functions at rest. Voluntary muscle activity refers to the additional energy expended during voluntary movements and exercises, such as walking or playing. Caloric increment (or the thermal effect of food) represents the energy used for the digestion, absorption, and metabolism of nutrients. Finally, thermogenesis involves the energy dissipated as heat during the maintenance of body temperature and in response to external stimuli, such as cold [1]. To maintain an ideal body weight (BW) and body condition score (BCS), veterinarians should recommend a precise daily intake of metabolizable energy (ME) for their patients [2,3,4]. Maintaining an ideal BW and BCS is essential, as being overweight and obese are associated with several comorbidities, such as orthopedic, cardiovascular, and metabolic diseases, in addition to reducing the quality and life expectancy of dogs. Conversely, malnutrition and low BW may indicate nutritional deficiencies, loss of muscle mass, and increased vulnerability to disease. Since ME reflects the energy available for use by the body after digestion, absorption, and the subsequent losses in urine and feces [5], it is the standard classification used in energy recommendations for dogs. However, the methods for determining ME expenditure are labor-intensive and difficult to apply in routine veterinary practice [6]. Consequently, predictive equations for canine energy requirements are recommended [7].

Kleiber [8] was the first to correlate daily energy expenditure with body weight across species and to develop an equation to predict the daily energy expenditure of a resting mammal. Fourteen years later, Kleiber [9] refined the formula and defined the resting energy requirement (RER) equation as follows: RER = 70 × BW^0.75^, where BW is body weight in kilograms and the result is given in kilocalories per day (kcal/d). The RER equation can be applied to any mammal, regardless of its weight and size, as energy requirements are more closely related to body surface area than to body weight [9,10]. Thus, BW raised to the 0.75 power is referred to as metabolic weight and is based on the relationship between body weight, body surface area, and energy requirements. In dogs, the exponent that best matches the relationship between BW and ME requirements has been widely debated, ranging from 0.64 to 0.88, before reaching a consensus at 0.75 [11].

The RER equation calculates the energy expenditure required to maintain the basal metabolic rate but does not account for voluntary muscle activity, caloric increment, or thermogenesis. To estimate the metabolizable energy requirements (MER), the factor 70 can be replaced with a value that reflects the dog’s current life stage [12]. In 2006, the National Research Council (NRC) published updated nutritional requirements for dogs and cats, recommending several life stage-specific predictive equations for MER, including MER = 95 × BW^0.75^ for inactive adult dogs and MER = 130 × BW^0.75^ for active adult dogs [11]. Most precursor studies on MER were conducted in laboratory or kennel dogs, whose MER was above 130 × BW^0.75^ [11,13,14]. More recent studies, such as that by Thes et al. [15], showed an average MER of 98 BW^0.75^ in healthy dogs based on data from 586 animals. Additionally, Madhusudhan et al. [16] observed a median MER of 103.4 × BW^0.75^ in German Shepherds and Labrador Retrievers. In a meta-analysis, Bermingham et al. [17] proposed a new allometric predictive equation, defined as 62.5 × BW^0.93^, which suggests higher MER values than those recommended by the NRC [11].

Energy requirements are influenced by factors such as age, breed, size, activity level, environment, and neutering status [13,14,17]. Neutering, for example, has been shown to alter the hormonal profile and reduce physical activity, leading to a decrease in daily energy expenditure in both male and female dogs [18]. Consequently, the literature suggests using the same predictive equation recommended for inactive dogs to estimate the MER of neutered dogs [11,19]. Additionally, despite hormonal and physiological differences between male and female dogs, no studies have demonstrated significant differences in daily energy requirements based on sex.

Given the numerous individual factors influencing MER, predictive equations may not always accurately reflect an animal’s actual energy expenditure. Their application should always be accompanied by weight monitoring and body condition scoring to allow for necessary adjustments. However, the occasional and generalized use of these equations, often incorporated into pet food labels, can mislead pet owners and contribute to energy imbalance. The high prevalence of overweight and obesity, affecting 40.5% to 55% of the canine population in Brazil and Japan, respectively [20,21], may, in part, be linked to inadequate energy recommendations derived from the occasional use of predictive equations [11,19]. Factors such as the feeding of treats, table scraps, and the amount of physical activity also influence the development of obesity. Therefore, given the importance of precise MER recommendations and ongoing efforts to refine predictive models [15,16,17], further research is needed to develop increasingly specific equations. Therefore, the hypothesis of this study is that the MER of dogs in clinical practice differs from the values typically recommended in the scientific literature. A broader and more comprehensive evaluation is necessary to better support the clinical decision-making process. Thus, this study aimed to determine the MER factor for dogs in clinical practice, taking into account variables such as sex, neutered status, body condition, muscle mass, body size, and age group. The findings will be compared with the recommended factors reported in the scientific literature.

## 2. Materials and Methods

### 2.1. Data Collection and Animal Classification

A retrospective evaluation was performed on 5086 medical records of dogs. These records were collected from the Veterinary Nutrition Service of the Veterinary Hospital, School of Veterinary Medicine and Animal Science, University of São Paulo, covering the period from 2013 to 2025.

The key data extracted from these records included age, sex, breed, BW, BCS, neutering status, MER, prescribed diet, daily food intake, and diagnosis. All information was gathered through a comprehensive clinical examination, which involved an oral interview and detailed anamnesis with the owners to establish a nutritional history, followed by a thorough physical examination. All procedures followed the guidelines established by Baldwin et al. [22] and were performed by trained veterinary staff.

For inclusion in the study, dogs needed to be over one year of age and have a BCS between 4 and 9 [23]. Critically, only records with consistent follow-up were selected, ensuring that body weight variations remained within ±5% over a 30-day period. During this entire period, the animals consumed the same food prescribed by the veterinarian during treatment. All selected dogs were categorized as inactive as a standardization criterion to estimate the initial MER, which was set at 95 kcal/kg of metabolic body weight (kg^0.75^), according to the European Federation of the Pet Food Industry (FEDIAF) recommendations for inactive dogs. Daily food allowances were meticulously adjusted to maintain body weight stability throughout the study. The actual energy intake (in kcal/day) was recorded based on the amount of food provided and its energy density as declared by the manufacturer. The energy factor for each dog was then calculated by dividing the daily energy intake by the animal’s metabolic body weight (BW^0.75^). These individual values were subsequently grouped and analyzed according to sex, neuter status, BCS, MMS, size, and life stage.

Records were excluded if they contained incomplete information, signs of hyporexia or anorexia, concomitant acute or chronic diseases, pregnancy, lactation, or if the dog was enrolled in a weight-loss program. Furthermore, records were discarded if the owners failed to adhere to the daily dietary recommendations or reported offering non-prescribed food or treats.

Regarding dietary management, all dogs were fed a commercially available diet. When the ME content was provided on the product label (which may have been estimated using different predictive equations at the manufacturer’s discretion), this value was used in the calculations. However, for diets without declared ME values, ME was estimated using the Atwater equations, as described by Marchi et al. 2025 [24].

Finally, based on comprehensive information in the medical records, the dogs were classified by the authors. These classifications included size (small ≤15 kg; medium >15 to 25 kg; large and giant >25 kg), life stage according to Creevy et al. [25] (young adult, mature adult, and senior), and body condition score according to Laflamme [23] (ideal, overweight, and obese).

### 2.2. Statistical Analysis

For the statistical analysis, all independent variables were coded as quantitative variables, with their levels in ascending order. Specifically, sex was coded as follows: female = 1, male = 2. Size was coded as follows: small = 1, medium = 2, large and giant = 3. Neutering status was coded as neutered = 1 and intact = 2. Body condition score was grouped and coded as follows: 4 or 5 = 1, 6 or 7 = 2, and 8 or 9 = 3. The muscle mass score was coded as follows: MMS 1 = 1, MMS 2 = 2, and MMS 3 = 3. Finally, life stage was coded as follows: young adult = 1, mature adult = 2, and senior = 3.

Initially, a General Linear Model (GLM) was applied using the energy factor as the dependent variable and six coded independent variables (sex, size, neutering status, BCS, MMS, and life stage). A forward selection procedure was employed, beginning with an intercept-only model and sequentially adding predictors that most significantly improved the model fit. Final model selection relied on the Schwarz Bayesian Information Criterion (SBC; Schwarz, 1978) [26], with stopping criteria further guided by the Akaike Information Criterion (AIC; Akaike, 1981) [27] and the predicted residual sum of squares (PRESS) statistic. The dataset was partitioned into training (*n* = 216), validation (*n* = 135), and test (*n* = 87) sets. The ultimate model was chosen based on the lowest SBC and average squared error (ASE) observed in the validation set (Table 1).

Subsequently, the energy factors were analyzed using a mixed linear model. This model incorporated the variables selected in the final step of the forward selection procedure (BCS and neutering status) as fixed effects, arranged in a 2 × 3 cross-classification. Combinations of sex, life stage, size, MMS, and source of ME value (labeled or Atwater) were included as random effects, along with the residual error. When significant effects were detected via the F-test, Tukey’s post-hoc test was applied for multiple comparisons. All analyses were performed using the PROC GLMSELECT and PROC MIXED procedures within the Statistical Analysis System software (version 9.4; SAS Institute Inc., Cary, NC, USA).

## 3. Results

**Figure 1:**
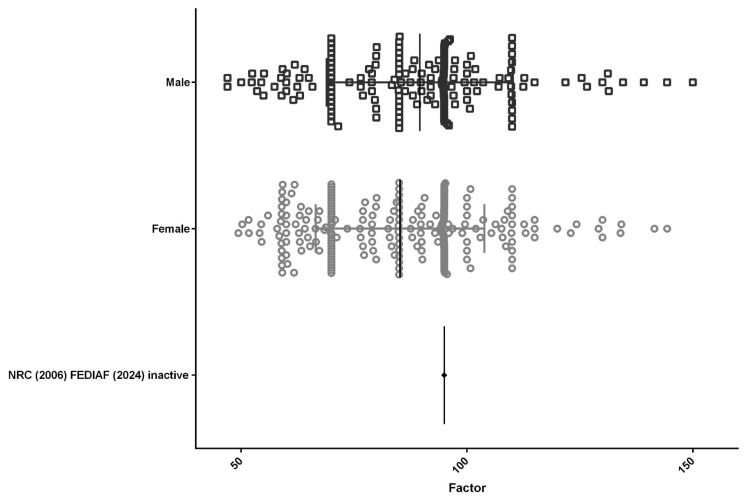
The population (*n* = 438) included 253 female dogs (57.8%) with a mean energy factor of 85.21 and 185 male dogs (42.2%) with a mean energy factor of 89.58.

**Figure 2:**
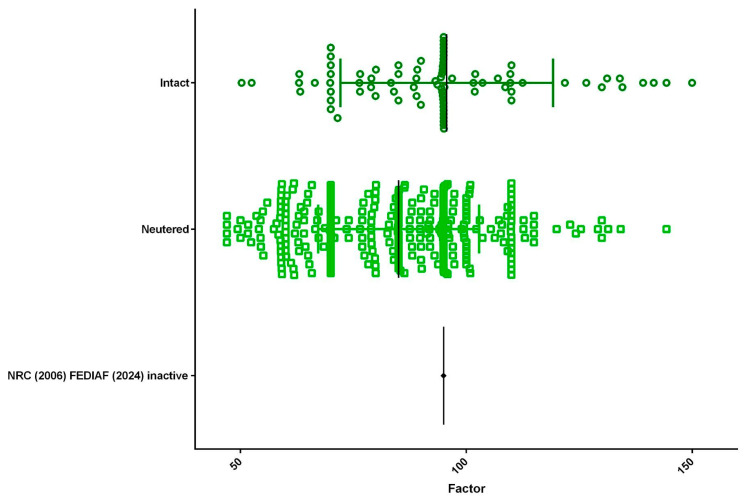
A total of 85 dogs (19.4%) were intact, with a mean energy factor of 95.68, and 353 dogs (80.6%) were neutered, with a mean energy factor of 84.98.

**Figure 3:**
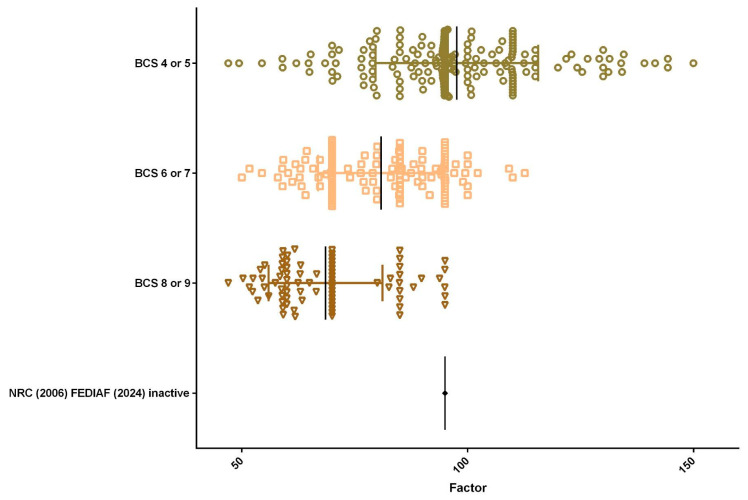
Body condition score distribution (*n* = 438). Half of the population (50.5%; *n* = 221) presented an ideal BCS (4 or 5), with a mean energy factor of 97.64. Additionally, 31.1% (*n* = 136) were overweight (BCS 6 or 7), with a mean energy factor of 80.87, and 18.5% (*n* = 81) were obese (BCS 8 or 9), with a mean energy factor of 68.58.

A total of 438 medical records met the criteria and were included in the data analysis. The population comprised 253 female (57.8%) and 185 male dogs (42.2%), with a mean energy factor of 85.21 for females and 89.58 for males (Figure 1). Regarding reproductive status, 353 dogs (80.6%) were neutered and 85 (19.4%) were intact, with mean energy factors of 84.98 and 95.68, respectively (Figure 2). As for BCS, half of the population (50.5%; *n* = 221) presented an ideal BCS (4 or 5), with a mean energy factor of 97.64. Additionally, 31.1% (*n* = 136) were classified as overweight (BCS 6 or 7), with a mean energy factor of 80.87, and 18.5% (*n* = 81) were obese (BCS 8 or 9), with a lower mean energy factor of 68.58 (Figure 3).

**Figure 4:**
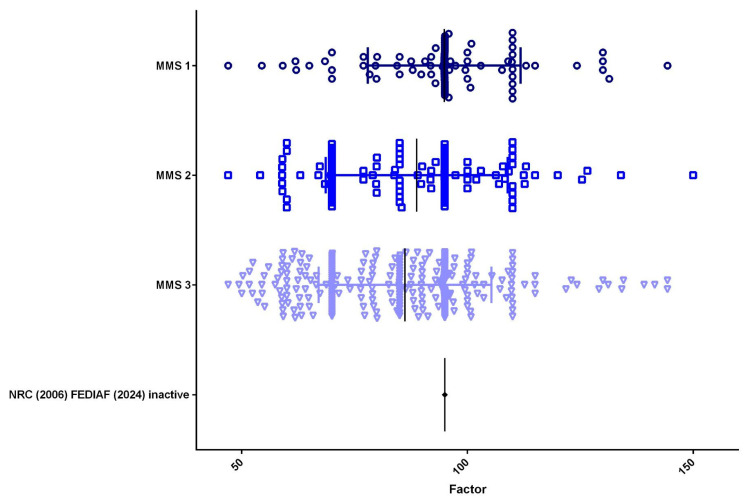
Distribution of muscle mass score (*n* = 438). Most dogs (70.8%, *n* = 310) exhibited normal muscle mass (MMS 3), with a mean energy factor of 86.20. Mild to moderate muscle loss (MMS 2) was observed in 24.9% of the dogs (*n* = 109), with a mean energy factor of 88.77, while 4.3% (*n* = 19) presented severe muscle loss (MMS 1), with a mean energy factor of 91.27.

Among the dogs, 221 (50.5%) had a BCS of 4 or 5, with a mean energy factor of 97.64; 136 (31.1%) had a BCS of 6 or 7, with a mean energy factor of 80.87; and 81 (18.5%) had a BCS of 8 or 9, with a mean energy factor of 68.58. The MMS assessment showed that 19 dogs (4.3%) had MMS 1, with a mean energy factor of 91.27; 109 dogs (24.9%) had MMS 2, with a mean energy factor of 88.77; and 310 dogs (70.8%) had MMS 3, with a mean energy factor of 86.20 (Figure 4).

**Figure 5:**
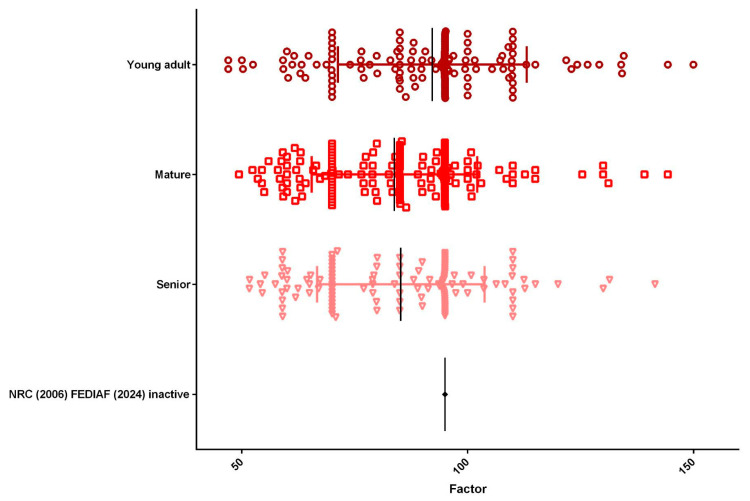
Life stage distribution (*n* = 438) was as follows: The population included 33.8% young adults (*n* = 148), with a mean energy factor of 92.20; 37.4% mature adults (*n* = 164), with a mean energy factor of 83.82; and 28.8% seniors (*n* = 126), with a mean energy factor of 85.22.

**Figure 6:**
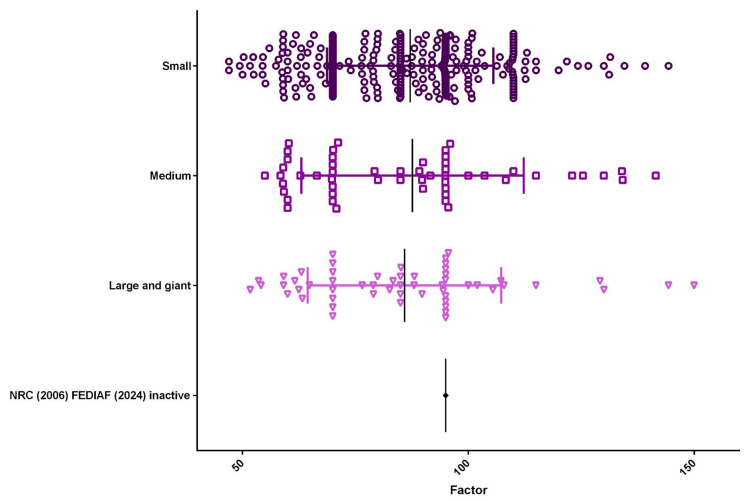
Size distribution (*n* = 438). Small-breed dogs represented 74.9% of the population (*n* = 328), with a mean energy factor of 87.16. Medium-breed dogs accounted for 12.3% (*n* = 54) of the sample, with a mean energy factor of 87.66, while large and giant breeds represented 12.8% (*n* = 56), with a mean energy factor of 85.88.

Based on life-stage classification, the population consisted of 148 young adults (33.8%), 164 mature adults (37.4%), and 126 seniors (28.8%) (Figure 5). The mean energy factor varied across life stages, with young adults showing the highest value (92.20), followed by seniors (85.22), and mature adults (83.82). Regarding body size, 328 (74.9%) dogs were classified as small breeds, 54 (12.3%) as medium breeds, and 56 (12.8%) as large or giant breeds (Figure 6). The mean energy factor was relatively similar across size categories: 87.16 for small breeds, 87.66 for medium breeds, and 85.88 for large and giant breeds.

Based on the forward selection procedure previously conducted, it was observed that the energy factor was directly influenced only by the neutering status and BCS of the dogs selected for this study (Table 1). Therefore, only these two variables were incorporated for further investigation, while the remaining variables were included as random effects to provide greater robustness to the statistical model. Based on this approach, a significant interaction between neutering status and BCS on the dogs’ energy factors was observed (*p* = 0.0089; Table 2).

Specifically, for dogs with a BCS of 4 or 5, the mean energy factor was 103.42 ± 2.21 kcal/kg for intact animals and 96.70 ± 1.85 kcal/kg for neutered animals. At a BCS of 6 or 7, the values were 81.34 ± 2.92 kcal/kg for intact dogs and 82.78 ± 1.65 kcal/kg for neutered dogs. For dogs with a BCS of 8 or 9, the mean energy factors were 66.90 ± 3.03 kcal/kg for intact and 70.13 ± 1.56 kcal/kg for neutered.

## 4. Discussion

Since the last NRC publication for dogs and cats in 2006, several studies have reported varying results regarding the equations established. When considering the clinical applicability of these equations in routine care, this variation may be even more pronounced due to multiple factors, including neutering, breed, sex, and the owner’s lifestyle, all of which can significantly influence a dog’s MER. In this context, it is important to note that ‘energy requirements’ refer to energy expressed per metabolic body weight (BW^0.75^), which allows for more accurate comparisons between animals of different sizes and body conditions. Neutering is a widely recommended procedure in the field of veterinary medicine. Trevejo et al. [28] estimated the prevalence of neutered dogs in the United States by analyzing 1,339,860 dog records from private veterinary hospitals in 2007. Their findings revealed that 64% of dogs were neutered, with females more likely to undergo the procedure than males. A later study conducted via an online questionnaire-based survey included responses from 2882 human participants representing 6400 dogs across seven different regions. These regions included the United Kingdom, France, North America (the United States and Canada), Germany, Northern Europe (Sweden, Norway, and Finland), Australia and New Zealand, Switzerland, and Austria. The results showed that national neutering rates ranged between 26% and 98% for males and 32% and 100% for females [29].

From a clinical perspective, a recent study conducted by O’Neill et al. [30] aimed to report the frequency of common disorders in dogs and explore the effects associated with neutering status, age, and sex of canine patients. A random sample of 22,333 dogs from 784 clinics was used. Demographic results demonstrated that females were more likely to be neutered (4856/10,540, 46.07%) than males (5241/11,718, 44.73%) (*p* = 0.044). Furthermore, neutered animals (71.66%) were more likely to have at least one recorded disorder than intact animals (61.17%, *p* < 0.001). The main disorder associated with neutered animals was obesity. In another study, Chiang et al. [31] reported a higher prevalence of overweight and obesity in neutered dogs (43.5% of 34,154 dogs) than in intact dogs (29.1% of 5884 dogs). Their findings also indicated that neutered dogs, females, and middle-aged or elderly dogs were at a higher risk of developing overweight and obesity.

The results of the present study, in line with previous clinical and epidemiological studies on the prevalence of obesity in dogs [20,21,32,33,34], demonstrate that spayed female dogs have a lower MER than the estimates provided by NRC [11] and FEDIAF [19]. This reduction in energy requirements may be partially explained by the decrease in energy expenditure following gonadectomy, as previously described in the literature [35]. Gonadectomy has also been associated with increased food intake and weight gain [36], potentially due to alterations in gonadal hormone regulation, which influences key peripheral hormones related to energy homeostasis, such as insulin and leptin [37]. These hormonal changes can disrupt the mechanisms responsible for signaling energy reserves to the central nervous system, contributing to both reduced energy expenditure and increased energy consumption.

In clinical practice, weight gain after dog neutering is commonly observed as a result of two main factors: increased ad libitum energy intake and decreased energy expenditure [36]. Schauf et al. [38] evaluated the effects of neutering and the primary energy-providing nutrients, fat and nonstructural carbohydrates, on food intake, blood concentrations of satiety-related hormones, and activity levels in dogs through a two-phase experiment. Twelve female Beagle dogs were assigned to either the control or the sterilization group. The authors found that neutering was not associated with inefficient control of food intake or significant variations in satiety hormone release in the gut (*p* > 0.10). However, it led to a reduced activity level compared with intact dogs (*p* < 0.05). Furthermore, the basal metabolic rate is directly linked to weight gain after neutering. Le Roux [39] evaluated ovariectomized female dogs fed a fixed amount of commercial food and observed a decrease in baseline metabolic rate following the procedure, along with a tendency to reduce overall energy expenditure [39].

According to the NRC [11] and FEDIAF [19], the predictive equation 95 × BW^0.75^ is recommended for calculating the MER of inactive dogs. However, these guidelines do not provide specific recommendations for neutered or inactive dogs. As a result, veterinarians often apply the equation for inactive dogs to estimate the nutritional needs of neutered dogs, considering their reduced energy demands. Despite this approach, traditional predictive equations, even those designed for inactive or neutered dogs, tend to overestimate energy requirements under the conditions observed in this study. Our findings indicate that smaller factors are necessary for neutered dogs, with recommended estimates of 82.24 kcal/day for neutered females and 82.49 kcal/day for neutered males. This result is highly relevant to clinical practices. Since the animals in our study had MER values below the lowest estimation equation, this may be linked to the high prevalence of overweight and obesity in dogs. Given that commercial pet food labels and veterinary nutritional recommendations are largely based on NRC and FEDIAF guidelines, overestimating energy requirements could contribute to excessive caloric intakes. When assessing the proportion of neutered dogs with MER below the recommended equation, we found that 47.12% of the evaluated dogs were in caloric excess.

According to the NRC [11] and FEDIAF [19], the predictive equation 95 × BW^0.75^ is recommended for calculating the MER of inactive dogs. However, these guidelines do not provide specific recommendations for neutered or inactive dogs. As a result, veterinarians often apply the equation for inactive dogs to estimate the nutritional needs of neutered dogs, considering their reduced energy demands. Despite this approach, traditional predictive equations, even those designed for inactive or neutered dogs, tend to overestimate energy requirements under the conditions observed in this study. Our findings demonstrate that neutering status and BCS significantly influenced MER. Neutered dogs consistently exhibited lower energy factors than intact dogs in the same BCS category. Specifically, intact dogs with an ideal BCS (4–5) had a mean MER factor of 103.42 kcal/BW^0.75^, while neutered dogs in the same condition had 96.70 kcal/BW^0.75^. As BCS increased, MER progressively decreased in both groups, with neutered dogs at BCS 8–9 presenting the lowest MER (66.90 kcal/BW^0.75^), followed by intact dogs at the same BCS (70.13 kcal/BW^0.75^). This interaction between neutering status and BCS (*p* = 0.0089) reinforces the need to individualize energy estimation. These results are highly relevant to clinical practice because the animals in our study exhibited MER values consistently below the lowest estimation proposed by the current guidelines. This may be directly associated with the high prevalence of overweight and obesity in the pet population. Given that commercial pet food labels and veterinary nutritional recommendations are largely based on NRC and FEDIAF guidelines, overestimating energy requirements could contribute to excessive caloric intakes. When assessing the proportion of neutered dogs with MER below the recommended equation, we found that 47.12% of the evaluated dogs were in caloric excess.

This result is consistent with the 40.5% prevalence of overweight and obese dogs recently estimated in São Paulo, Brazil, by Porsani et al. [21]. Although obesity is not widely recognized as a disease by dog owners, scientific literature classifies it as a significant endocrine disorder in dogs [40,41,42]. In general, obesity is a multifactorial condition primarily caused by an imbalance between excessive energy intake and reduced energy expenditure [43,44]. Clinically, obese dogs are in a state of chronic low-grade inflammation, characterized by elevated concentrations of pro-inflammatory cytokines, hormonal dysregulation, and increased immune cell recruitment [18,45]. Additionally, obesity predisposes dogs to various health issues, including orthopedic disorders [46,47], cardiovascular diseases [48,49,50], respiratory conditions [51,52], cancer [53], and metabolic disorders, such as insulin resistance [54] and hyperlipidemia [42].

Additionally, the owner’s lifestyle plays a crucial role in a pet’s physical condition, particularly in sedentary households with excessive food consumption habits. Since dogs are entirely dependent on their owners for both food and physical activity, this impact is significant [55]. To date, little is known about the intensity of physical exercise in dogs, regardless of whether they are at an ideal weight, are overweight, or are obese. One study reported that obese dogs receive less exercise than non-obese dogs, according to owner-reported data [56]. However, the type and intensity of physical exercise were not assessed, and self-reported exercise levels are often subject to response bias, which can lead to overestimation or underestimation of physical activity levels [57]. A bidirectional relationship may exist between physical exercise, energy expenditure, and obesity. Reduced physical activity leads to a lower MER, which, when combined with excessive food intake, can contribute to obesity based on the estimated energy expenditure [58]. Thus, it is necessary to reassess the use of the term “active”, as it may cause confusion when making nutritional recommendations for dogs that are physically active but not engaged in athletic activities. Kour et al. [59] reported that 57.14% of obese dogs were never exercised and were significantly more likely to be obese (odds ratio: 29.467), a finding consistent with other studies [34,41,60].

Despite the European Federation of the Pet Food Industry publishing an updated version of its Nutritional Guidelines for dogs and cats in 2024, which includes revised predictive equations for different activity levels, the present study focused exclusively on inactive dogs. Therefore, it was not possible to assess whether the equations for active animals, such as those for moderate (110 × BW^0.75^), high (125–175 × BW^0.75^), or extreme (860–1240 × BW^0.75^) activity, are appropriate. Nevertheless, our results strongly suggest that even the current equation recommended for inactive dogs (95 × BW^0.75^) tends to overestimate the MER in the clinical population we evaluated. This finding highlights a significant limitation in the current nutritional guidelines, which do not differentiate between intact and neutered animals nor fully consider the high prevalence of overweight and obesity observed in pet dogs. While further research involving active dogs is necessary to evaluate the adequacy of these higher energy factors, our data indicate that the predictive equation for inactive animals may be outdated for the general pet population. Therefore, adjustments are needed to better reflect the actual energy requirements of companion dogs, particularly those that are neutered and sedentary.

One limitation of this study is the variability in the sources of ME values used in the dataset. While the majority of ME data (approximately 88%) was estimated using Atwater predictive equations [24], a smaller portion (around 12%), mostly older records, relied on labeled ME values provided by pet food manufacturers. To mitigate potential interference in data variability, the source of ME was included as a random effect in our model. The choice to incorporate diverse data sources reflects the reality of clinical practice, where nutritionists and veterinarians must often rely on manufacturer-reported ME values when predictive data are unavailable.

## 5. Conclusions

This study highlights the substantial variability in the metabolizable energy requirements of dogs in clinical settings, revealing important limitations in the exclusive use of standardized energy recommendations. The significant interaction observed between neutering status and body condition reinforces the complexity of the factors influencing energy needs and emphasizes the importance of individualized nutritional assessment.

## Figures and Tables

**Table 1:** Forward selection summary for the selection of independent variables used in the analysis of the energy factor, showing the average values.

Step	Effect Entered	Effects on the Model	AIC	SBC	PRESS	ASE	Validation ASE	Test ASE
0	Intercept	1	1521.21	1306.58	90,098.86	413.27	379.69	316.88
1	Body condition score	2	1444.87	1237.00	63,071.01	284.92	225.78	216.29
2	Neutering status	3	1441.34	1236.84 *	62,504.57	277.71	224.66 *	224.86
3	Sex	4	1439.80	1238.67	62,037.64	273.19	227.15	230.01
4	Muscle mass score	5	1437.76	1243.39	62,156.70	265.66	231.93	250.47
5	Size	6	1436.33	1248.70	61,997.01	259.06	236.86	261.71
6	Life stage	7	1436.43	1255.90	63,955.83	254.84	236.25	251.70

* Optimal value of criterion (SBC and validation ASE); selection stopped at a local minimum of the AIC criterion; ASE = average squared error.

**Table 2:** Estimates of means for energy factors according to neutering status (NS) and body condition score (BCS) (*n* = 438).

NS	BCS						*p*-Value		
	BCS 4 or 5	SEM	BCS 6 or 7	SEM	BCS 8 or 9	SEM	NS	BCS	Interaction NS × BCS
Intact	103.42 ^A; a^	2.21	81.34 ^A; b^	2.92	66.90 ^A; c^	3.03	<0.0001	0.9178	0.0128
Neutered	96.70 ^B; a^	1.85	82.78 ^A; b^	1.65	70.13 ^A; c^	1.56			

SEM = standard error of the mean; ^A–B^ Different uppercase letters in the same column indicate a significant difference in neutering status within the same body condition score; ^a–c^ Different lowercase letters in the same row indicate a significant difference in body condition score for the same reproductive status.

## Data Availability

The original contributions presented in this study are included in this article. Further inquiries should be directed to the corresponding author.

## Abbreviations

The following abbreviations are used in this manuscript:

BCS	Body condition score
BW	Body weight
ME	Metabolizable energy
FEDIAF	European Federation of the Pet Food Industry
MER	Maintenance energy requirement
NRC	National Research Council
RER	Resting energy requirement

## References

[1] Burger I.H. (1994). Energy needs of companion animals: Matching food intakes to requirements throughout the life cycle. J. Nutr..

[2] Laflamme D.P. (2006). Understanding and managing obesity in dogs and cats. Vet. Clin. N. Am. Small Anim. Pract..

[3] Blaxter K.L. (1989). Energy Metabolism in Animals and Man.

[4] Debraekeleer J., Gross K.L., Zicker S.C., Hand M.S., Thatcher C.D., Remillard R.L., Roudebush P., Novotny B.J. (2010). Feeding mature adult dogs: Middle aged and older. Small Animal Clinical Nutrition.

[5] Hall K.D., Heymsfield S.B., Kemnitz J.W., Klein S., Schoeller D.A., Speakman J.R. (2012). Energy balance and its components: Implications for body weight regulation. Am. J. Clin. Nutr..

[6] Hill R.C. (2006). Challenges in measuring energy expenditure in companion animals: A clinician’s perspective. J. Nutr..

[7] Walker R.N., Heuberger R.A. (2009). Predictive equations for energy needs for the critically ill. Respir. Care.

[8] Kleiber M. (1947). Body size and metabolic rate. Physiol. Rev..

[9] Kleiber M. (1961). The Fire of Life: An Introduction to Animal Energetics.

[10] Blaxter K.L. (1964). Metabolism and Metabolic Body Size: A Study with Cattle and Sheep.

[11] NRC (2006). Nutrient Requirements of Dogs and Cats.

[12] Hand M.S., Thatcher C.D., Remillard R.L. (2010). Small animal clinical nutrition: An iterative process. Small Animal Clinical Nutrition.

[13] Kienzle E., Rainbird A. (1991). Maintenance energy requirement of dogs: What is the correct value for the calculation of metabolic body weight in dogs?. J. Nutr..

[14] Finke M.D. (1994). Energy requirements of adult female beagles. J. Nutr..

[15] Thes M., Koeber N., Fritz J., Wendel F., Dillitzer N., Dobenecker B., Kienzle E. (2016). Metabolizable energy intake of client-owned adult dogs. J. Anim. Physiol. Anim. Nutr..

[16] Madhusudhan H.S., Chandrapal Singh K., Krishnamoorthy U., Umesh K.G., Butterwick R., Wrigglesworth D. (2018). Estimation of maintenance energy requirements in German shepherd and Labrador retriever dogs in Bangalore, India. J. Anim. Physiol. Anim. Nutr..

[17] Bermingham E.N., Thomas D.G., Cave N.J., Morris P.J., Butterwick R.F., German A.J. (2014). Energy requirements of adult dogs: A meta-analysis. PLoS ONE.

[18] Vendramini T.H., Amaral A.R., Pedrinelli V., Zafalon R.V., Rodrigues R.B., Brunetto M.A. (2020). Neutering in dogs and cats: Current scientific evidence and importance of adequate nutritional management. Nutr. Res. Rev..

[19] FEDIAF (2024). Nutritional Guidelines for Complete and Complementary Pet Food for Cats and Dogs.

[20] Usui S., Yasuda H., Koketsu Y. (2016). Characteristics of obese or overweight dogs visiting private Japanese veterinary clinics. Asian Pac. J. Trop. Biomed..

[21] Porsani M.Y.H., Teixeira F.A., Oliveira V.V., Pedrinelli V., Dias R.A., German A.J., Brunetto M.A. (2020). Prevalence of canine obesity in the city of São Paulo, Brazil. Sci. Rep..

[22] Baldwin K., Bartges J., Buffington T., Freeman L.M., Grabow M., Legred J., Ostwald D. (2010). AAHA Nutritional assessment guidelines for dogs and cats. J. Am. Anim. Hosp. Assoc..

[23] Laflamme D.P. (1997). Development and Validation of a Body Condition Score System for Dogs. Canine Pract..

[24] Marchi P.H., Amaral A.R., Príncipe L.D.A., Risolia L.W., Rentas M.F., Fasolai A.B., Zafalon R.V.A., Finardi G.L.F., Jeremias J.T., Pedreira R.S. (2025). Accuracy of Predictive Equations for Metabolizable Energy Compared to Energy Content of Foods for Dogs and Cats Estimated by In Vivo Methods in Brazil. Animals.

[25] Creevy K.E., Grady J., Little S.E., Moore G.E., Strickler B.G., Thompson S., Webb J.A. (2019). 2019 AAHA Canine Life Stage Guidelines. J. Am. Anim. Hosp. Assoc..

[26] Schwarz G. (1978). Estimating the Dimension of a Model. Ann. Stat..

[27] Akaike H. (1981). Likelihood of a Model and Information Criteria. J. Econom..

[28] Trevejo R., Yang M., Lund E.M. (2011). Epidemiology of surgical castration of dogs and cats in the United States. J. Am. Vet. Med. Assoc..

[29] Berthoud D., Nevison C., Waterhouse J., Hawkins D. (2011). The prevalence of neutered pet dogs (*Canis familiaris*) across countries of the western world. J. Vet. Behav..

[30] O’Neill D.G., James H., Brodbelt D.C., Church D.B., Pegram C. (2021). Prevalence of commonly diagnosed disorders in UK dogs under primary veterinary care: Results and applications. BMC Vet. Res..

[31] Chiang C.F., Villaverde C., Chang W.C., Fascetti A.J., Larsen J.A. (2022). Prevalence, risk factors, and disease associations of overweight and obesity in dogs that visited the veterinary medical teaching hospital at the University of California, Davis from January 2006 to December 2015. Top. Companion Anim. Med..

[32] McGreevy P.D., Thomson P.C., Pride C., Fawcett A., Grassi T., Jones B. (2005). Prevalence of obesity in dogs examined by Australian veterinary practices and the risk factors involved. Vet. Rec..

[33] Mao J., Xia Z., Chen J., Yu J. (2013). Prevalence and risk factors for canine obesity surveyed in veterinary practices in Beijing, China. Prev. Vet. Med..

[34] Gates M.C., Zito S., Harvey L.C., Dale A., Walker J.K. (2019). Assessing obesity in adult dogs and cats presenting for routine vaccination appointments in the North Island of New Zealand using electronic medical records data. N. Z. Vet. J..

[35] Kanchuk M.L., Backus R.C., Morris J.G., Rogers Q.R., Calvert C.C. (2003). Weight gain in gonadectomized normal and lipoprotein lipase–deficient male domestic cats results from increased food intake and not decreased energy expenditure. J. Nutr..

[36] De Godoy M.R. (2018). Pancosma comparative gut physiology symposium: All about appetite regulation: Effects of diet and gonadal steroids on appetite regulation and food intake of companion animals. J. Anim. Sci..

[37] Mystkowski P., Schwartz M.W. (2000). Gonadal steroids and energy homeostasis in the leptin era. Nutrition.

[38] Schauf S., Salas-Mani A., Torre C., Bosch G., Swarts H., Castrillo C. (2016). Effect of Sterilization and of Dietary Fat and Carbohydrate Content on Food Intake, Activity Level, and Blood Satiety–Related Hormones in Female Dogs. J. Anim. Sci..

[39] Le Roux P.H. (1983). Thyroid status, oestradiol level, work performance and body mass of ovariectomised bitches and bitches bearing ovarian autotransplants in the stomach wall. J. S. Afr. Vet. Assoc..

[40] Eisele I., Wood I.S., German A.J., Hunter L., Trayhurn P. (2005). Adipokine gene expression in dog adipose tissues and dog white adipocytes differentiated in primary culture. Horm. Metab. Res..

[41] German A. (2010). Obesity in companion animals. Practice.

[42] Brunetto M.A., Nogueira S., Sá F.C., Peixoto M., Vasconcellos R.S., Ferraudo A.J., Carciofi A.C. (2011). Correspondência entre obesidade e hiperlipidemia em cães. Ciênc. Rural.

[43] McCrory M.A., Fuss P.J., Saltzman E., Roberts S.B. (2000). Dietary determinants of energy intake and weight regulation in healthy adults. J. Nutr..

[44] Osto M., Lutz T.A. (2015). Translational value of animal models of obesity—Focus on dogs and cats. Eur. J. Pharmacol..

[45] Piantedosi D., Palatucci A.T., Giovazzino A., Ruggiero G., Rubino V., Musco N., Cortese L. (2020). Effect of a weight loss program on biochemical and immunological profile, serum leptin levels, and cardiovascular parameters in obese dogs. Front. Vet. Sci..

[46] Kealy R.D., Lawler D.F., Ballam J.M., Lust G., Smith G.K., Biery D.N., Olsson S.E. (1997). Five-year longitudinal study on limited food consumption and development of osteoarthritis in coxofemoral joints of dogs. J. Am. Vet. Med. Assoc..

[47] Kealy R.D., Lawler D.F., Ballam J.M., Mantz S.L., Biery D.N., Greeley E.H., Stowe H.D. (2002). Effects of diet restriction on life span and age-related changes in dogs. J. Am. Vet. Med. Assoc..

[48] Pereira-Neto G.B., Brunetto M.A., Champion T., Ortiz E.M., Carciofi A.C., Camacho A.A. (2014). Avaliação da pressão arterial sistêmica em cães obesos: Comparação entre os métodos oscilométrico e doppler ultrassônico. Pesq. Vet. Bras..

[49] Piantedosi D., Di Loria A., Guccione J., De Rosa A., Fabbri S., Cortese L., Ciaramella P. (2016). Serum biochemistry profile, inflammatory cytokines, adipokines and cardiovascular findings in obese dogs. Vet. J..

[50] Tropf M., Nelson O.L., Lee P.M., Weng H.Y. (2017). Cardiac and metabolic variables in obese dogs. J. Vet. Intern. Med..

[51] Devito F.C., Patricio G.C.F., Flôr P.B., Vendramini T.H.A., Amaral A.R., Pfrimer K., Cortopassi S.R.G. (2020). Comparative study of anaesthesia induction in obese dogs using propofol dosages based on lean body weight or total body weight. Vet. Anim. Sci..

[52] Pereira-Neto G.B., Brunetto M.A., Oba P.M., Champion T., Villaverde C., Vendramini T.H., Camacho A.A. (2018). Weight loss improves arterial blood gases and respiratory parameters in obese dogs. J. Anim. Physiol. Anim. Nutr..

[53] Marchi P.H., Vendramini T.H., Perini M.P., Zafalon R.V., Amaral A.R., Ochamotto V.A., Brunetto M.A. (2022). Obesity, inflammation, and cancer in dogs: Review and perspectives. Front. Vet. Sci..

[54] Brunetto M.A., Sá F.C., Nogueira S.P., Gomes M.D.O.S., Pinarel A.G., Jeremias J.T., Carciofi A.C. (2011). The intravenous glucose tolerance and postprandial glucose tests may present different responses in the evaluation of obese dogs. Br. J. Nutr..

[55] Gille S., Fischer H., Lindåse S., Palmqvist L., Lärka J., Wolf S., Söder J. (2023). Dog owners’ perceptions of canine body composition and effect of standardized education for dog owners on body condition assessment of their own dogs. Vet. Sci..

[56] Courcier E.A., Thomson R.M., Mellor D.J., Yam P.S. (2010). An epidemiological study of environmental factors associated with canine obesity. J. Small Anim. Pract..

[57] Sirard J.R., Pate R.R. (2001). Physical activity assessment in children and adolescents. Sports Med..

[58] Ekelund U., Brage S., Besson H., Sharp S., Wareham N.J. (2008). Time spent being sedentary and weight gain in healthy adults: Reverse or bidirectional causality?. Am. J. Clin. Nutr..

[59] Kour H., Agrawal R., Singh R., Pande N. (2019). Prevalence and risk factors for obesity in dogs. J. Pharm. Innov..

[60] Bland I.M., Guthrie-Jones A., Taylor R.D., Hill J. (2009). Dog obesity: Owner attitudes and behaviour. Prev. Vet. Med..

